# Comment on “The Prevalence of Skilled Birth Attendant Utilization and Its Correlates in North West Ethiopia”

**DOI:** 10.1155/2015/379836

**Published:** 2015-12-13

**Authors:** Siddharudha Shivalli, Soujanya Kaup

**Affiliations:** ^1^Department of Community Medicine, Yenepoya Medical College, Yenepoya University, Mangalore 575018, Karnataka, India; ^2^Department of Ophthalmology, Yenepoya Medical College, Yenepoya University, Mangalore 575018, Karnataka, India

Alemayehu and Mekonnen [1] reported the prevalence of Skilled Birth Attendant Utilization and its correlates in North West Ethiopia. Authors' efforts are commendable. This study provides valuable information for the evidence based fine tuning of present reproductive and child health strategies in the study area. However, the following issues and concerns need to be addressed.

Introduction part of the paper should be written in such a way that even a reader without subject expertise should be able to understand the present situation and need of the study [2]. In this paper, authors have nicely elaborated the present scenario about the skilled birth attendance utilization in the study area and stressed on the need of the study. However, keeping the international readers in mind, a word about the following would have been more informative: present strategies/programmes in Ethiopia to enhance the skilled birth utilization, role of Health Services Extension Workers (HSEWs), and the operational definition of a “kebele.”

It is highly advisable to follow the STROBE (Strengthening the Reporting of Observational Studies in Epidemiology) checklist while reporting cross-sectional studies [3]. The STROBE Statement is endorsed by a number of biomedical journals.

Authors have justified the sample size and clearly delineated the multistage random sampling adopted in this study. They mention that the study mothers were randomly selected from the rosters of HSEWs. It appears that HSEWs keep a record of all the births irrespective of the place of birth in their area. The definition of “skilled birth attendant” should have been explicitly mentioned in Methods. In addition, reliability of records and measures taken to identify the missed cases from the records should have been stated. According to us, inclusion criterion should be any woman who delivered in the last year irrespective of the outcome (abortion/still birth/live birth) and place of delivery.

While exploring the correlates of Skilled Birth Attendant Utilization, a brainstorming of all the possible factors which are likely to influence in deciding the place of delivery should be done. In this study, authors did consider key demographic and obstetric correlates. However, we believe that the authors have missed certain key factors such as knowledge risk factors/danger signs in pregnancy, discussion with spouse/family member about the place of delivery, bad obstetric history/bad experiences, and decision maker in the family [4, 5].

Epidemiological studies extrapolate the results from the study sample on study population. Hence, outcome variable should be reported with 95% confidence intervals [3]. In this study, the prevalence of Skilled Birth Attendant Utilization should have been reported as 18.7% (*n* = 70, 95% CI: 15.1–23.1). Regression analysis is an excellent way of adjusting the effect of confounders. However, it is highly advisable to assess and report the adequacy of applied regression model. Failure to do so may lead to misleading or incorrect inferences. Although the study sample was relatively large, a word about *R* ^2^ (explaining the variance in the Skilled Birth Attendant Utilization) of the applied regression model would have been more affirmative.

According to STROBE guidelines, one has to discuss limitations of the study, taking into account sources of potential bias or imprecision [3]. The associations observed in this cross-sectional study may not imply causality. In addition, there is a risk of recall bias as women who had delivered in 12 months prior to the study were included.

Nonetheless, we must congratulate the authors for investigating an important public health issue on a large scale.
